# Transcriptomic insights into antagonistic effects of gibberellin and abscisic acid on petal growth in *Gerbera hybrida*

**DOI:** 10.3389/fpls.2015.00168

**Published:** 2015-03-17

**Authors:** Lingfei Li, Wenbin Zhang, Lili Zhang, Na Li, Jianzong Peng, Yaqin Wang, Chunmei Zhong, Yuping Yang, Shulan Sun, Shan Liang, Xiaojing Wang

**Affiliations:** Guangdong Provincial Key Lab of Biotechnology for Plant Development, College of Life Sciences, South China Normal UniversityGuangzhou, China

**Keywords:** abscisic acid, antagonistic regulation, *Gerbera hybrida*, gibberellin, petal growth, RNA-seq

## Abstract

Petal growth is central to floral morphogenesis, but the underlying genetic basis of petal growth regulation is yet to be elucidated. In this study, we found that the basal region of the ray floret petals of *Gerbera hybrida* was the most sensitive to treatment with the phytohormones gibberellin (GA) and abscisic acid (ABA), which regulate cell expansion during petal growth in an antagonistic manner. To screen for differentially expressed genes (DEGs) and key regulators with potentially important roles in petal growth regulation by GA or/and ABA, the RNA-seq technique was employed. Differences in global transcription in petals were observed in response to GA and ABA and target genes antagonistically regulated by the two hormones were identified. Moreover, we also identified the pathways associated with the regulation of petal growth after application of either GA or ABA. Genes relating to the antagonistic GA and ABA regulation of petal growth showed distinct patterns, with genes encoding transcription factors (TFs) being active during the early stage (2 h) of treatment, while genes from the “apoptosis” and “cell wall organization” categories were expressed at later stages (12 h). In summary, we present the first study of global expression patterns of hormone-regulated transcripts in *G. hybrida* petals; this dataset will be instrumental in revealing the genetic networks that govern petal morphogenesis and provides a new theoretical basis and novel gene resources for ornamental plant breeding.

## Introduction

Petals are a particularly important component of the reproductive system of higher plants. As well as protecting the stamen and pistil, petals are instrumental in attracting the correct pollinator(s) to ensure successful pollination, which depends on their specific size, shape, color and arrangement (Glover and Martin, 1998). These unique characteristics are established during petal morphogenesis and are fundamentally connected with the identity, growth and development of petal primordium (Krizek and Fletcher, 2005; Alvarez-Buylla et al., 2010).

A number of gene regulatory networks (GRNs) that govern petal development, together with the associated transcription factors (TFs), have been identified recently (Alvarez-Buylla et al., 2010; O'Maoileidigh et al., 2014). The ABCDE model indicates that petal identity is determined by the combined actions of class A (*AP1*), B (*AP3* and *PI*), and E (*SEP*) genes, whereas petal growth is negatively regulated by class C (*AGAMOUS, AG*) genes (Krizek and Fletcher, 2005; Alvarez-Buylla et al., 2010). In early flower primordium formation, *AINTEGUMENTA* (*ANT*), *JAGGED* (*JAG*), and *ARGOS* (an *A*uxin-*R*egulated *G*ene involved in *O*rgan *S*ize) function as positive regulators of cell proliferation (Krizek, 1999; Dinneny et al., 2004; Ohno et al., 2004). By contrast, Big Brother (*BB*), *KLU* and *DA1* are repressors of cell division in the flower (Disch et al., 2006; Anastasiou et al., 2007; Li et al., 2008). The *AtNAP* gene was shown to function at the transition point between cell division and cell expansion in *Arabidopsis thaliana* petals and stamens, acting downstream of class B genes (*AP3/PI*) (Sablowski and Meyerowitz, 1998). In the later stages of petal growth, *BIGPETAL* (*BPEp*), a basic helix-loop-helix (bHLH) TF, is known to regulate petal size in *A. thaliana* by restricting cell expansion (Szecsi et al., 2006).

Phytohormones are well-known mediators of flower organ morphogenesis. In *A. thaliana*, auxin regulates many aspects of floral growth (Aloni et al., 2006) and the auxin response factor 8 (ARF8) interacts with BPEp to modulate cell expansion in petals (Varaud et al., 2011). Moreover, mutation that affect jasmonic acid (JA) biosynthesis leads to reduced petal growth (Brioudes et al., 2009) and JA regulates the expression of *BPEp*, suggesting that *BPEp* may also have a role in JA-mediated petal growth (Brioudes et al., 2009; Varaud et al., 2011). Furthermore, ARF6 and ARF8 induce the production of JA to promote the growth of petals and stamen by triggering the expression of *MYB21* and *MYB24* (Reeves et al., 2012). Therefore, auxin and JA function coordinately in the regulation of petal growth in *A. thaliana*, representing a very close association within the GRN involved (Varaud et al., 2011).

It is known that gibberellin (GA) regulates many critical biological events in plants, including seed germination, stem elongation and flowering (Sun, 2008; Hedden and Thomas, 2012). Recent evidence has revealed that GA signaling is crucial to petal growth (Sun, 2008). As a versatile regulator, abscisic acid (ABA) has been shown to act antagonistically to the function of GA in a variety of developmental processes, including floral transition and fruit development (Razem et al., 2006). However, it is unknown whether such an antagonistic relationship exists in the regulation of petal growth. In addition, in contrast to the significant progress made in elucidating the GRN involving auxin and JA that governs petal growth (Brioudes et al., 2009; Varaud et al., 2011), the GRN associated with GA and ABA remains poorly understood.

*Gerbera hybrida*, a member of the sunflower family, is emerging as a model for the investigation of the genetic regulation of organ growth and development (Kotilainen et al., 1999; Laitinen et al., 2007; Zhang et al., 2012). The ray petals in *G. hybrida* only exhibit substantial cell expansion after stage 3 when the proliferation-to-expansion phase transition occurs (Meng and Wang, 2004; Laitinen et al., 2007), and can serve as a useful system for investigation of the regulatory network governing cell expansion. Previously, we presented a morphological description and the cellular basis of the ray petal growth in *G. hybrida*, thereby establishing the necessary groundwork for the molecular characterization of petal growth (Meng and Wang, 2004; Zhang et al., 2012). In the current study, we used powerful second-generation sequencing technology to determine the transcriptome of the ray petals of *G. hybrida* at stage 3. This allowed us to produce high-resolution digital profiles of global gene expression relating to petal growth, thereby revealing the GRN that underpins the antagonistic control of petal growth by GA/ABA signaling. Since samples were collected from well-characterized stages and tissues, the transcriptome data are highly conducive to cross-lab or cross-species comparisons. In addition, the initial analysis of the wealth of molecular information has generated unprecedented molecular insights into petal growth.

## Materials and methods

### Plant material and growth conditions

*G. hybrida* “Shenzhen No. 5” seedlings were grown in a greenhouse at Zengcheng Ornamental Center (Guangzhou, China) as described by Zhang et al. (2012) at a temperature of 26/18°C (day/night) and relative humidity of 65–80%. The development stages of the inflorescence were defined according to Meng and Wang (2004). Inflorescences at stage 1.5 (between stages 1 and 2), which are approximately 1.5 cm in diameter with a ray petal (petal) length of 6 mm, were used for the *in vivo* experiment. For the *in vitro* experiment, petals at stage 3 were used.

### Hormone and inhibitor treatments

For the evaluation of petal length as described below, GA and/or ABA treatments were employed in *in vivo* or *in vitro* experiments, depending on the purpose of the analysis. Five to six inflorescences of similar size were included for each treatment. *In vivo* treatments were performed by spraying the stage 1.5 inflorescences with 3–5 ml 10 μM GA_3_ or 50 μM ABA once a day; inflorescences were sampled after 9 days. As a control, inflorescences sprayed with 0.1% ethanol in deionized water were sampled in parallel. *In vitro* treatments were in accordance with the previously described procedures (Huang et al., 2008; Zhang et al., 2012) using stage 3 inflorescences. Briefly, about 10 petals of the outermost whorl of ray flowers were detached from the inflorescences, placed on two layers of Whatman filter paper soaked in 3% sucrose solution, with or without hormones (10 μM GA_3_ or 50 μM ABA) as supplementary elements, and treated for 9 days. Ten or more petals were used in the *in vitro* experiments; the duration of treatment varied depending on the purpose of the assay performed, as indicated below.

To evaluate the interaction between the effects of GA and ABA, we performed *in vitro* experiments using a combination of hormones, in which, for example, after preculturing the petals with GA for 2, 12, or 24 h, ABA was added to the medium, with the final measurements being made after 72 h. Conversely, where ABA was the initial hormone used, GA was added during the experiment.

The widely used inhibitors of GA and ABA biosynthesis (White et al., 2000; Kusumoto et al., 2006; Martinez-Andujar et al., 2011; Hedden and Thomas, 2012), paclobutrazol (PAC) and fluridone (FLU), were also used in this study. In the *in vitro* experiments described above, the phytohormones were replaced by PAC (10 μM) or FLU (0.1 μM).

Hormones and inhibitors were acquired from Sigma-Aldrich Chemical Co. (Shanghai, China). Both *in vivo* and *in vitro* experiments were each replicated at least three times.

### Measurement of petal and cell length

To measure petal elongation, whole petals from each *in vivo* experiment were harvested and images of the petals were scanned using an EPSON-G850A scanner (EPSON, China) and photographed. Measurement of petal length was performed using Image J software (http://rsb.info.nih.gov/ij/, NIH, MD, USA). In total, more than six inflorescences were collected for each treatment and the lengths of 10 petals from each inflorescence were measured. Data from at least 60 petals were thus averaged to estimate the petal length under each treatment condition.

Elongation of three petal regions, namely top, middle or basal, was also recorded for *in vitro* treated petals. The lengths of three regions of the same petals before and after treatment were measured. For each individual measurement, a total of 10 petals were used and three independent measurements were made. The elongation rates were calculated according to the equation: Elongation rate = (L*t*–L*i*)/L*i* × 100%, where L*t* is the petal length after treatment, while L*i* is the initial length of each petal before treatment. Data from individual measurements were averaged.

For measurement of cell length, petals were sampled after *in vitro* treatments. A 1 mm^2^ petal block was dissected from the center of each of the top, middle and basal regions, and was stained by immersion in 0.1 mg mL^−1^ propidium iodide for 5 min at 25°C, followed by rinsing thoroughly with deionized water to remove excess stain solution, before flattening samples on a glass slide. Abaxial epidermal cell images were obtained with a laser confocal scanning microscope (LSM710/ConfoCor2, Carl-Zeiss, Jena, Germany), after which the length of individual cells was measured using Image J software. From at least 10 petals detached from different inflorescences, more than 100 cells were randomly selected for length measurement, which was performed before and after treatment. Untreated samples gave the initial length, L*i*, while samples after treatment provided L*t* values. The elongation rate was estimated using the equation described above and data from three independent measurements of biological replicate samples were averaged.

After measurement, One-Way ANOVA was conducted to test for statistical significance using SPSS 13.0 (SPSS Inc., Chicago, IL, USA). Duncan's test was applied to assess the differences between treatments.

### RNA-seq

**Figure 1:**
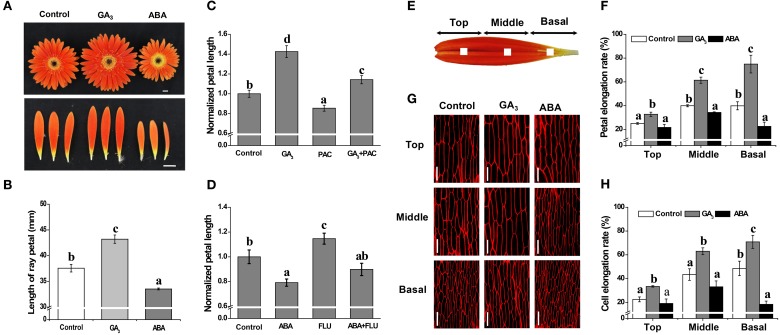
**Antagonistic effects of GA and ABA on growth of petals in *G. hybrida***. *G. hybrida* was grown in a greenhouse under the conditions described in Materials and Methods. Plants with inflorescences at stage 1.5 were sprayed with deionized water (with 0.1% ethanol) (Control), 10 μM GA_3_ or 50 μM ABA and were subjected to morphological characterization **(A)** and petal length measurement **(B)** after 9 days of treatment. Sixty or more ray petals were measured for each treatment and the value are given as average lengths ± SE. Representative examples of inhibition of GA- or ABA-derived effects on petal growth by PAC (10 μM) or FLU (0.1 μM) are shown in **(C,D)**, for which the control petal length was set as 1. One mm^2^ blocks at the center of the top, middle or basal regions of the petal are indicated in **(E)**. Detached petals treated with Control, 10 μM GA_3_ or 50 μM ABA for 9 days were used for morphological characterization of adaxial epidermal cell using a confocal microscope **(G)** and measurement of elongation rate of each petal region (**F**, *n* = 10) or cell (**H**, *n* > 100). Three biological replicates were performed for each measurement. Values are given as mean ± SE. Letters above the bars indicate significant differences between the respective values (*p* < 0.05). Scale bar represents 1 cm **(A)** or 50 μm **(G)**.

Before treatment with GA or ABA, petals were precultured on 3% sucrose medium (pH 5.8) for 2 days (Huang et al., 2008). Petals were treated for either 2 or 12 h with GA or ABA. At least 200 petals from 20 inflorescences at stage 3 were used for each combination of phytohormone and treatment duration. Petals cultured on 3% sucrose solution without addition of phytohormone were used as the control. The entire basal regions of petals (Figure 1E) were collected and pooled for each treatment combination. Total RNA was extracted from each basal pool, resulting in six samples, corresponding to the six treatment combinations of each hormone and treatment duration; these were denoted Control2h, Control12h, GA2h, GA12h, ABA2h, and ABA12h. TRIzol® reagent (Invitrogen, USA) was used for total RNA extraction according to the manufacturer's instructions. DNase I (Takara, Japan) was used to remove genomic DNA. The quality of total RNA was checked with an Agilent 2100 Bioanalyzer (Agilent Technologies, Palo Alto, CA). Those samples with an RNA integrity number (RIN) > 8 were used to prepare cDNA libraries, as previously described (Kuang et al., 2013). The libraries were used for paired-end 45 × 2 sequencing using Illumina HiSeq™ 2000 at the Beijing Genomics Institute (BGI) (Shenzhen, China). In total, six sets of raw reads were obtained, corresponding to Control2h, Control12h, GA2h, GA12h, ABA2h, and ABA12h. All sequence data were deposited at the NCBI in the Short Read Archieve (SRA) database under the accession numbers SRX850776, SRX850779, SRX850784, SRX850787, SRX850789, and SRX850790 for Control2h, GA2h, ABA2h, Control12h, GA12h, and ABA12h, respectively.

### Data processing and analysis

Raw read processing and primary bioinformatics analysis of the transcript datasets were conducted at Genedenovo Biotechnology Co., Ltd (Guangzhou, China). In brief, raw reads was filtered to remove “dirty” data, including adaptor sequences, the reads in which unkown bases are greater than 10% and low-quality reads containing more than 50% bases with *Q* ≤ 5. The clean reads thus generated were mapped to the previously assembled *G. hybrida* transcriptome (Kuang et al., 2013) using SOAPaligner/soap2 (Li et al., 2009). Mismatches of no more than two bases were allowed, with separate alignments being performed for each sample independently. Unigenes mapped by at least one read, in at least one sample, were identified for further analysis. Estimation of gene expression and identification of differentially expressed genes (DEGs) were conducted using a modified method of that described previously (Audic and Claverie, 1997). Transcript abundance was expressed as RPKM (reads mapped per 1000 bp per million sequenced reads) (Mortazavi et al., 2008). RPKM values presenting as “0” were artificially set to “0.001” for subsequent analysis. Comparisons of RPKM between treatments (treatment2h vs. Control2h, treatment 12h vs. Control12h, treatment12h vs. treatment2h) were performed for each Unigene. Those with a fold-change of ≥2 and a false discovery rate (FDR) < 0.001, in at least one comparison, were considered as significant DEGs.

DEGs were subjected to enrichment analysis for both KEGG pathway and GO annotation terms. Before KEGG pathway analysis, KEGG Orthology terms for DEGs were retrieved from the KEGG pathway database (http://www.genome.jp/kegg/). The enrichment analysis was performed by comparing the observed DEG count to the expected count of the genes involved in a given pathway with a random distribution of the previously reported transcriptome (Kuang et al., 2013). A hypergeometric test was performed for statistical analysis and the *p*-value cut off was 0.05. For GO enrichment analysis, Gene Ontology (GO) annotations for each DEG were retrieved by mapping to GO terms in the database at http://www.geneontology.org. For DEGs with opposite regulation patterns, GO terms were also retrieved according to the annotations of *A. thaliana* homologs at http://www.arabidopsis.org, followed by performing GO enrichment analysis using the BinGO App in Cytoscape 3.2.0 (http://cytoscape.org/) against the whole *A. thaliana* genome. GO terms for Biological Processes (GO-BP) with a FDR ≥0.05 were considered significant. Hierarchical clustering analysis was performed using MeV 4.9.0 (http://www.tm4.org/mev.html) by considering the RPKM value as the normalized transcript level for a given gene.

### Quantitative real time PCR (qRT-PCR) validation

Total RNA was extracted from the basal region of petals (Figure 1E) using TRIzol® Reagent (Invitrogen, USA) according to the manufacturer's instructions and quantified with a NanoDrop 1000 Spectrophotometer (Fisher Rochester, NY, USA). Two μg RNA was treated with DNase I (Takara, Japan) according to the manufacturer's instructions, followed by cDNA synthesis using the SuperScript® III First-Strand Synthesis System (Invitrogen, USA) in a 40 μl total reaction volume with Random Primer 6 (Takara, Japan). For qRT-PCR, transcripts of target genes were amplified in a 20 μl reaction containing 2 μl cDNA (corresponding to 20 ng RNA), 1 μl primers and 5 μl SsoFast™ EvaGreen® Supermix (Bio-Rad, USA). Quantitation of each transcript was repeated using total RNA from three independent samples as starting materials and each qPCR was performed in triplicate. The primers are listed in Supplemental Table S1. Expression levels of the tested genes were normalized to that of the *ACTIN* (AJ763915) gene as previously described (Kuang et al., 2013).

## Results

### Effect of GA and ABA on petal growth

The *in vivo* experiments performed with intact inflorescences (stage 1.5) revealed that, compared with the average petal length of 37.6 mm in the control, GA promotes elongation of the petal to an average length of 43.2 mm, whereas ABA treatment results in petals that are shorter than controls, with an average length of 33.3 mm. Thus, GA treatment produces a significant increase in petal length, while ABA treatment produces a significant decrease (Figures 1A,B; *p* < 0.01), i.e., the two phytohormones have opposite effects on inflorescence size. We also found that treatment with PAC or FLU can suppress or enhance petal elongation (Figures 1C,D; *p* < 0.05), respectively. Moreover, PAC-mediated suppression and FLU-mediated enhancement of petal length can be reversed by the application of exogenous GA and ABA (Figures 1C,D), respectively. We interpret these data as illustrating that GA and ABA have contrasting effects on petal elongation.

The *in vitro* experiments showed that 9 days of GA treatment significantly increased the elongation rate of petal tissue in the top, middle and basal regions by 33, 61, and 75%, respectively, compared to increases of 25, 40, and 40% in the control for the same regions (Figure 1F; *p* < 0.05). Elongation rates following ABA treatment, however, were 22, 34, and 23%, respectively, indicating a significant inhibition in the middle and basal regions (Figure 1F; *p* < 0.05). We further showed that cell elongation rates were greatly increased in the presence of GA, by 33, 63, and 71% in the top, middle and basal regions, respectively, but only in the basal region was cell elongation rate significantly suppressed by ABA treatment (an increase of only 18% vs. a 49% increase in the control) (Figures 1G,H; *p* < 0.05). These results indicate that petal elongation is associated with cell elongation, and that the antagonistic effects of GA and ABA are predominantly limited to the basal region.

**Figure 2:**
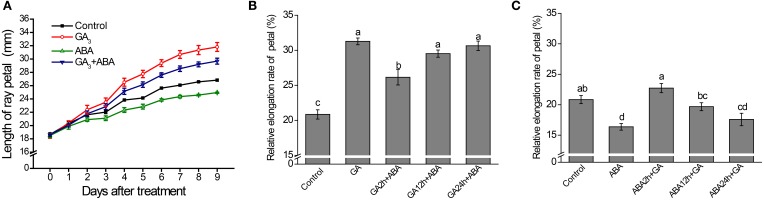
**Effects of GAor ABA on petal growth are attenuated by co-application of the hormones**. Petals of inflorescences at stage 3 were detached and placed on filter paper soaked with 3% sucrose (Control) or with hormone supplementation and were cultured as described in the text. **(A)** Time-course dynamics of petal length under control conditions or after treatment with 10 μM GA_3_, 50 μM ABA or a mixture of 10 μM GA_3_ and 50 μM ABA. A total of 10 petals for each treatment were cultured for 9 days. **(B)** Effect on petal elongation rate of addition of ABA after pre-culture with GA alone for 2, 12, and 24 h. **(C)** Effect on petal elongation rate of addition of GA after pre-culture with ABA alone for 2, 12, and 24 h. Petals were cultured for a total of 72 h. Control: petals were continuously cultured with 3% sucrose; GA: petals were continuously cultured with 3% sucrose plus GAalone; ABA: petals were continuously cultured with 3% sucrose plus ABA alone; GA+ABA: petals were continuously cultured with a mixture of GA and ABA; GA2h+ABA/ABA2h+GA: petals were pre-treated with GA or ABA for 2 h followed by addition of ABA or GA. In other cases, the duration of GA or ABA pre-culture before ABA or GA supplementation was as indicated. Each value is the mean ± SE (*n* = 6 petals). The experiment was repeated at least three times with similar results. Representative data are presented. Letters above the bars in **(B,C)** indicate significant differences between the respective values (*p* < 0.05).

The combined effects of GA and ABA on petal elongation were further tested *in vitro*. The growth dynamics of the petal indicated that GA-mediated promotion and ABA-mediated repression of petal elongation could both be attenuated by the co-application of ABA and GA, respectively, suggesting that the effects of GA and ABA are antagonistic (Figure 2A). Interestingly, the promotion of petal elongation by GA was only significantly attenuated when ABA was applied within 2 h of the initial GA treatment: no significant attenuation in petal length was seen if ABA was added at 12 or 24 h (Figure 2B). Moreover, ABA-mediated repression of petal growth was overcome by supplementation with GA 2 or 12 h after the initial application of ABA (Figure 2C).

### Effect of GA and ABA on the petal transcriptome

**Figure 3:**
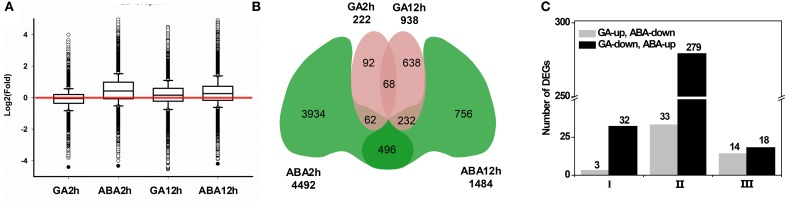
**Global comparisons of transcript profiles and DEGs in response to GA and ABA treatments**. Petals of inflorescences at stage 3 were detached, placed on filter papers soaked with 3% sucrose (Control) or with hormone supplementation and were cultured as described in the text. Basal regions of the petals were collected after 2 or 12 h of culture, followed by total RNA extraction for the subsequent RNA-Seq analyses and data mining. **(A)** Global transcript profiles at the 2 or 12 h time point after culturing with hormone supplementation as indicated. Each box plot shows the distribution of the relative transcription level [log_2_(fold-change)] of genes with at least one read mapped to the *G. hybrida* transcriptome in one sample. The red line indicates a one-fold change relative to the control transcription level. **(B)** Venn diagram for DEGs identified at each time point of hormone application as indicated. **(C)** Number of DEGs antagonistically regulated by GA and ABA. Class I and III correspond to the DEGs after 2 and 12 h of hormone application, respectively, and Class II was identified by comparing 12 h samples with 2 h samples.

An investigation of the GA/ABA-associated GRN that modulates petal growth was performed using RNA-seq data. After removing contaminated and low-quality sequences, all reads were mapped onto the published transcriptome, which contains 47,104 Unigenes (Kuang et al., 2013). Unigenes represented by at least one mapped read were accepted for subsequent analyses. In total, we generated 42,773 Unigenes and the coverage for individual RNA samples ranged from 76 to 87% (Table 1). The global distribution of the relative expression level, which is determined by a log2-transformed fold-change relative to the control, is shown in Figure 3A. ABA treatment for 2 h resulted in greater variation of the relative expression level, with the distribution ranging from −4.34 to 5.11 and a higher mean value of 0.46, whereas GA treatment for 2 h gave a distribution range from −4.41 to 3.98 and a mean of −0.08. At the 12 h time point, the range of transcription levels was spread more broadly, from −6.24 to 4.49 and −4.20 to 7.38 for GA and ABA treatment, respectively. The mean transcription level after 12 h, however, decreased to 0.30 with ABA treatment, but increased to 0.18 with GA treatment.

**Table 1:** **Summary of the mapping reads and Unigenes identified by RNA-seq**.

	**Number of Unigenes**
Total Unigenes (Kuang et al., 2013)	47,104
Total mapped Unigenes	42,773
**MAPPED IN SAMPLE**	
Control2h	39,503 (84%^*^)
GA2h	38,824 (82%^*^)
ABA2h	40,767 (87%^*^)
Control12h	35,788 (76%^*^)
GA12h	36,438 (77%^*^)
ABA12h	35,928 (76%^*^)

* *Percent of Unigenes with at least one mapped read*.

### Identification of DEGs compatible with antagonistic effects of GA and ABA

We then screened the DEGs from the collection of 42,773 Unigenes (Table 1). Using the criteria of fold-change ≥2 and FDR < 0.001, we identified 222 and 938 DEGs after GA treatment for 2 and 12 h, respectively, of which 68 Unigenes were common in both datasets (Figure 3B). There were 4492 DEGs in response to ABA treatment for 2 h (Figure 3B), which is 20-fold higher than the response to GA treatment for the same time. After treatment with ABA for 12 h, 1484 DEGs were identified, of which 496 DEGs also occurred in the group treated with ABA for 2 h (Figure 3B; Supplemental Tables S2–S5). Notably, with increasing duration of treatment, the number of DEGs in response to GA treatment increased, but decreased in response to ABA. In total, we obtained a set of 6278 DEGs for the subsequent identification of genes involved in the antagonistic regulation of petal growth by GA and ABA.

We further analyzed those DEGs antagonistically regulated by GA and ABA. Firstly, we defined three classes of DEGs. Class I and Class III DEGs refer to those antagonistically regulated by GA and ABA after 2 and 12 h treatment, respectively. We found that, relative to control (Control2h), three DEGs up-regulated by GA (GU) were shown to be down-regulated by ABA (AD) after 2 h treatment, in contrast to 32 DEGs down-regulated by GA (GD) but up-regulated by ABA (AU) (Figure 3C; I). Thus, a total of 35 DEGs (57.38% of the DEGs that co-regulated by GA and ABA at 2 h) were antagonistically regulated by GA and ABA at 2 h (Figure 3C; Supplemental Tables S6, S7). When the hormone treatments were extended to 12 h, the number of antagonistically regulated Class III DEGs was nearly the same (Figure 3C; III). Although the number of Class III DEGs (32) was similar to that of Class I DEGs, the ratio was decreased to 13.8% (Figure 3C; Supplemental Tables S6, S7). Class II DEGs, which were identified by comparing the datasets representing treatment for 12 h and treatment for 2 h, had an opposite pattern of change between GA and ABA treatment during the test period. For Class II DEGs, we identified 312 DEGs showing such an opposite dynamic change from 2 to 12 h, among which 33 DEGs showed a GU/AD pattern, whereas 279 DEGs showed a GD/AU pattern (Figure 3C; Supplemental Tables S8, S9).

**Figure 4:**
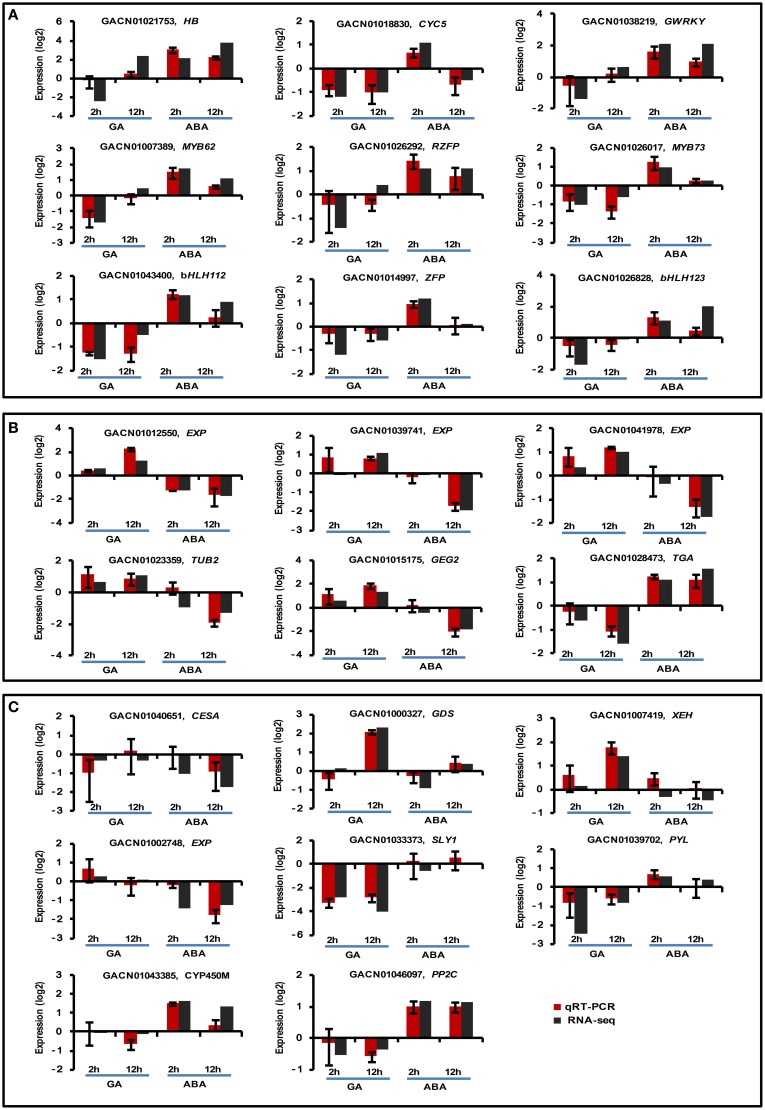
**Real-time quantitative PCR validation of transcript profiles for a subset of DEGs**. Twenty-three DEGs were selected for real-time qPCR validation, including nine Class I **(A)**, six Class III **(B)**, and eight randomly selected DEGs **(C)**. *G. hybrida ACTIN* (AJ763915) was used as the normalization control. Sample collection was conducted as described in the text. Three biological repeats were included for each condition. The y-axis indicates the expression of each DEG under the denoted conditions relative to control by log2-transformed RQ value for qPCR or fold-change value for RNA-seq. Error bars indicate the interval between the log2-transformed values of maximum or minimum RQ.

Twenty-three DEGs were selected for qRT-PCR. Specifically, eight DEGs were randomly selected (Figure 4C), nine DEGs were selected from Class I (Figure 4A) and six were selected from Class III (Figure 4B). Overall, the qRT-PCR data showed patterns similar to those obtained from RNA-Seq for these DEGs, although the particular values of fold-change were different.

### Enrichment analysis of DEGs antagonistically regulated by GA and ABA

To retrieve GO annotations for DEGs, we searched the TAIR 10 protein database using BlastX; GO annotations were assigned to each DEG according to the best hit. Enrichment analysis of DEGs was performed using BinGO with the default setting of FDR < 0.05, and compared with the *A. thaliana* whole genome GO annotation. By separately analyzing the three DEG classes, we observed that the GO-Biological Process (GO-BP) termed “cell wall organization” was overrepresented at 12 h within the set of Class III DEGs having a GU/AD pattern (Table 2). The sub-category “cell wall loosening” indicates the specific processes involved. For Class II DEGs with a GU/AD pattern, the GO-BP termed “apoptosis” was overrepresented (Table 2). Overrepresented categories were not found for Class I DEGs with a GU/AD pattern, but genes with the opposite pattern, GD/AU, involved in “regulation of transcription”, were mostly enriched in the 2 h dataset of Class I DEGs (Table 2).

**Table 2:** **DEGs with enriched GO terms**.

**Unigene ID**	**Description**	**GA2h**	**GA12h**	**ABA2h**	**ABA12h**
CELL WALL ORGANIZATION/CELL WALL LOOSENING (III)^*^					
GACN01006243	Expansin	−0.91	**−2.42**	0.61	**1.05**
GACN01012550	Expansin	0.59	**1.27**	**−1.28**	**−1.76**
GACN01038094	Expansin	0.77	**1.32**	**−1.55**	**−1.99**
GACN01039741	Expansin	0.10	**1.09**	−0.07	**−1.94**
GACN01041978	Expansin	0.34	**1.02**	−0.33	**−1.74**
GACN01023151	Expansin	0.81	**2.16**	**−1.07**	**−1.90**
APOPTOSIS (II)^*^					
GACN01018625	BCL-2-associated athanogene 5	0.30	**1.68**	**1.69**	−0.62
GACN01031726	Probable disease resistance protein	0.20	**3.26**	**1.44**	**−7.36**
GACN01015754	TIR-NBS-LRR class disease resistance protein	−0.83	0.32	**3.21**	**1.12**
GACN01011634	TIR-NBS-LRR class disease resistance protein	−0.11	**1.25**	**2.32**	**1.21**
GACN01026830	RPM1 interacting protein 13	−0.15	**1.09**	**3.46**	0.38
GACN01022318	NB-ARC domain-containing disease resistance protein	**−1.00**	**1.68**	**2.09**	0.97
GACN01023277	Putative TIR-NBS-LRR class disease resistance protein	**−2.64**	**1.09**	**1.85**	**−1.20**
GACN01009587	NB-ARC domain-containing disease resistance	−0.70	0.42	**1.33**	0.19
GACN01043665	Putative TIR-NBS-LRR class disease resistance protein	0.17	**1.19**	**2.74**	0.16
GACN01017010	Disease resistance protein RGC2	**−1.70**	−0.35	**2.05**	−0.53
REGULATION OF TRANSCRIPTION (I)^*^					
GACN01007389	MYB domain protein 62 like	**−1.73**	0.48	**1.70**	**1.05**
GACN01014997	Transcription factor zinc finger protein	**−1.16**	−0.62	**1.18**	0.14
GACN01026828	Transcription factor bHLH123	**−1.66**	−0.08	**1.13**	**2.02**
GACN01018830	Cycloidea-like 5	**−1.18**	**−1.02**	**1.13**	−0.49
GACN01038219	WRKY DNA-binding protein 30	**−1.35**	0.59	**2.11**	**2.06**
GACN01021753	Homeobox-leucine zipper protein AtHB-7	**−2.41**	**2.42**	**2.18**	**3.76**
GACN01026017	MYB domain protein 73	**−1.02**	−0.64	**1.04**	0.33
GACN01043400	Transcription factor bHLH112-like	**−1.47**	−0.47	**1.24**	0.87
GACN01026292	Ring zinc finger protein	**−1.39**	0.40	**1.11**	**1.05**

* *The bracketed Roman numeral indicates the DEG class under consideration. Fold changes greater than 2 relative to the corresponding control are in bold*.

KEGG pathway enrichment analysis was also carried out to elucidate the interaction of GA/ABA mediated pathways in petal growth. Of the ~47,000 Unigenes in *G. hybrida* (Kuang et al., 2013), 20,483 can be annotated and mapped to different pathways (data not shown). Not surprisingly, transcripts encoding proteins involved in plant hormone signal transduction were significantly enriched in all samples (Table 3), showing that 24 of the 103 annotated DEGs in the GA2h treatment were associated with these pathways. In addition, GA and ABA also regulated the expression of genes involved in multiple hormone signaling pathways (Supplemental Table S10). Moreover, we also identified crosstalk between the biosynthesis and metabolism pathways of multiple hormones. For example, genes involved in diterpenoid biosynthesis, which is associated with the gibberellin biosynthesis and metabolism pathway (Sun, 2008), were significantly enriched by both GA and ABA treatment. Similarly, the carotenoid biosynthesis pathway, which contributes to ABA biosynthesis and metabolism, was overrepresented after 12 h GAtreatment. Interestingly, genes involved in the biosynthesis of zeatin, a class of cytokinin (CK), were identified after 2 h GA or ABA treatment, suggesting crosstalk between the metabolic pathways of the three hormones.

**Table 3:** **Pathways differentially regulated by GA and ABA**.

**Pathway**	**GA2h**	**GA12h**	**ABA2h**	**ABA12h**
Plant hormone signal transduction	24 (6.8)^**^	46 (29.7)^**^	165 (146.2)^*^	78 (54.2)^**^
Plant-pathogen interaction	11 (6.6)	38 (29.0)	239 (143.0)^**^	91 (53.0)^*^
Phosphatidylinositol signaling system	–	5 (3.8)	38 (20.7)^**^	8 (7)
Cyanoamino acid metabolism	5 (0.8)^**^	7 (3.3)^*^	14 (15.7)	17 (6.3)^**^
Starch and sucrose metabolism	1 (2.3)	20 (10.3)^**^	51 (50.8)	40 (18.8)^**^
Diterpenoid biosynthesis	0 (0.3)	10 (1.1)^**^	17 (5.6)^**^	3 (2.1)
Carotenoid biosynthesis	1 (0.6)	8 (2.8)^**^	18 (14.0)	7 (5.2)
Photosynthesis—antenna proteins	0 (0.2)	1 (0.8)	2 (3.8)	16 (1.4)^**^
Flavonoid biosynthesis	1 (0.9)	17 (3.8)^**^	22 (18.9)	11 (7.0)
Anthocyanin biosynthesis	1 (0.0)	0 (0.3)	1 (1.3)	4 (0.5)^**^
ABC transporters	2 (0.8)	2 (3.6)	27 (17.6)^*^	15 (6.5)^**^
Zeatin biosynthesis	3 (0.8)^*^	5 (3.4)	28(16.7)^**^	8 (6.2)

*The number of DEGs in the corresponding pathway is shown. Expected counts from a random distribution based on the statistical analysis described by Nemhauser et al. (2006) are shown in parenthesis. The p-value was calculated using Fisher's Exact Test. Numbers significantly different to controls are shown in bold*.

* and

** *indicate p < 0.05 and p < 0.01, respectively*.

### Transcript profiling of DEGs antagonistically regulated by GA and ABA

**Figure 5:**
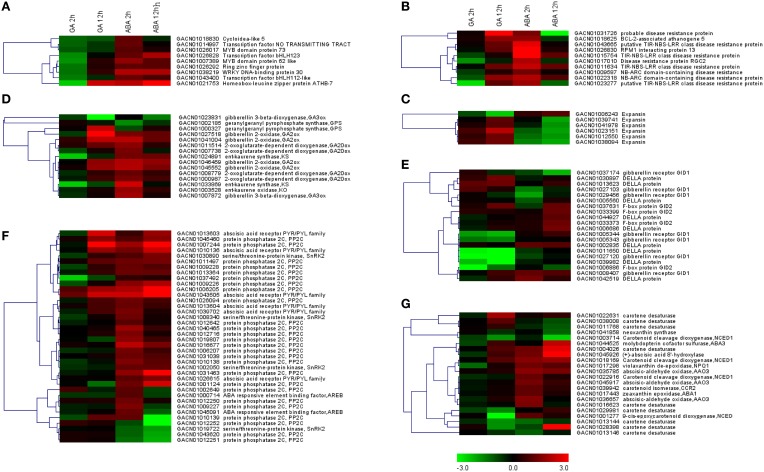
**Transcript profiles of genes involving in enriched GO categories or KEGG pathways**. Log2-transformed fold-change values were used for clustering analysis in MeV, which represent the relative transcription level under the denoted conditions relative to the control. **(A)** DEGs associated with regulation of transcription. **(B,C)** DEGs with the GO terms of “cell death” or “cell wall organization.” **(D–G)** Unigenes involved in pathways related to gibberellin biosynthesis **(D)** or signaling **(E)**, or ABA biosynthesis **(G)** or signaling **(F)**.

The three sets of DEGs annotated “regulation of transcription,” “cell wall organization” or “apoptosis” were loaded separately into MeV for hierarchical clustering analysis. It was apparent that “regulation of transcription” genes were repressed at the 2 h time point under GA treatment conditions; however, they were gradually activated from 2 to 12 h (Figure 5A; Table 2). ABA treatment promoted transcription of all these genes at 2 h, but most of them were repressed from 2 to 12 h. Five of the six genes involved in “cell wall organization” were significantly activated from 2 to 12 h with GA treatment, indicating constitutive activation by GA. ABA led to a slight increase in transcript abundance at the 2 h point, but reduced transcript levels at 12 h (Figure 5C; Table 2). The DEGs grouped under “apoptosis” all presented GU/AD patterns from 2 to 12 h (Figure 5B; Table 2).

Genes involved in biosynthesis, metabolism and signaling pathways associated with GA and ABA were retrieved from the DEG set according to the KEGG annotation. Clustering analysis indicated that a large proportion of these DEGs exhibited opposite expression patterns at 2 h of GA vs. ABA treatment, but a somewhat similar pattern at 12 h (Figures 5D–G). For example, four putative members of the gibberellin 2-oxidase (GA2ox) gene family exhibited GD/AU patterns. Genes encoding ent-kaurene synthase (KS) and ent-kaurene oxidase (KO), which initially showed the GD/AU expression pattern at 2 h, were up-regulated by GA but repressed by ABA from 2 to 12 h (Figure 5D). Gibberellin 3-oxidase which contribute to the generation of active GAs, were regulated by GA and ABA in a similar manner (Figure 5D). However, two genes (GACN01003714, GACN01001277) for NCED (9-cis-epoxy-carotenoid dioxygenase) homologs, encoding the rate limiting enzyme in ABA biosynthesis (Thompson et al., 2000), displayed GD patterns (Figure 5G).

## Discussion

### GA and ABA differentially modulate petal growth

Petal development is related to cell division and cell expansion, in which many phytohormones and genes are involved (Alvarez-Buylla et al., 2010; Krizek and Anderson, 2013). Previous studies have shown that at stage 3 of petal development in *G. hybrida*, the petal size is mainly determined by cell expansion, and not by cell division (Meng and Wang, 2004; Laitinen et al., 2007; Zhang et al., 2012). Two findings in our current study extend these conclusions, showing that GA and ABA have antagonistic effects on petal growth by modulation of cell elongation at the basal region: (1) petal/cell elongation is enhanced by GA but repressed by ABA when each phytohormone is applied alone (Figure 1); (2) the increase in petal length by GA and the reduction in petal length by ABA are attenuated by the co-application of ABA and GA, respectively (Figure 2).

Further evidence at the transcriptional level demonstrates that GA and ABA have different and contrasting effects on global transcription profiles in petal, especially after 2 h treatment. Thus, the number of DEGs identified at 2 h following ABA treatment was higher than after GA treatment (Figure 3), similar to the findings obtained in *A. thaliana* (Nemhauser et al., 2006). Treatment for 12 h, however, did not result in dramatic differences, implying that time-course expression changes of many genes occurred from 2 to 12 h. These opposing effects on transcription were further backed by analyses of the DEGs and the corresponding transcript profiles in response to GA or ABA treatment. It was found that the number of Class II DEGs (Supplemental Tables S8, S9) was greater than that of Class I or III (Figure 3C). Furthermore, while most Class II DEGs showed an elevation in transcript abundance between 2 and 12 h during GA treatment, they showed the opposite trend when treated with ABA. As expected, genes involved in pathways associated with the metabolism of diverse hormones were affected by both phytohormones. Taken together, these observations suggest that GA and ABA perturb various GRNs, resulting in antagonistic effects on petal growth.

### DEGs associated with hormone pathways are enriched by GA and ABA treatment

Hormones have been reported to play a role in petal development in several plants. GA levels transitorily increase in *Gaillardia* petals at the start of the corolla's fast growth stage, then decrease later on (Koning, 1985). In an *Arabidopsis* GA-deficient mutant, petal growth is arrested, but the defect is completely eliminated by application of GA (Goto and Pharis, 1999). A tomato GA-deficient mutant initiates flower buds, but floral development is not completed unless the mutant is treated with GA (Jacobsen and Olszewski, 1991). ABA, on the other hand, is usually associated with petal senescence, and accumulates to high levels in senescent rose petals (Kumar et al., 2008). Silencing a homeodomain-leucine zipper I transcription factor gene in rose delays ABA-induced petal senescence (Lü et al., 2014). In the current study, we demonstrated that GA treatment promotes, and ABA treatment suppresses, petal growth in *G. hybrida*. When the biosynthesis of endogenous GA and ABA are inhibited by PAC and FLU, respectively, the effects of GA and ABA treatment are reversed. These results suggest that the perturbation of endogenous GA and ABA biosynthesis in *G. hybrida* partly contributes to the antagonistic action of these two hormones on petal growth.

Our RNA-seq data also support the above hypothesis, showing that DEGs involved in hormone-associated pathways, including GA or ABA biosynthesis and signaling, are oppositely perturbed by GA or ABA treatment for 2 h. However, these differences are no longer apparent when the treatment is extended to 12 h (Figures 5D–G), indicating that transcriptional regulation of hormone-associated pathways is an early and transient event during petal growth. We interpret our results to further suggest that inhibition by GA and ABA of each other's effects on petal growth could result from at least two scenarios: (1) GA and ABA could target the same genes. For example, *KS* and *KO*, which contribute to GA biosynthesis (Sun, 2008), are regulated by both GA and ABA (Figure 5D). In addition, genes for TPR (tetratricopeptide) repeat-containing protein (GACN01042178), CBL-interacting protein kinase (GACN01010590) and leucine-rich repeat transmembrane protein kinase (GACN01030993), which are annotated in both GA- and ABA-associated pathways (Van der Knaap et al., 1999; Rosado et al., 2006; Pandey et al., 2008), are also perturbed by both GA and ABA (Supplemental Tables S6, S8); (2) GA and ABA interfere with each other's biosynthetic or signaling pathways by an effect on the components of these pathways. For example, *GA2ox* proteins, which play roles in converting active GAs to inactive forms (Sun, 2008), are activated by ABA, suggesting that ABA treatment could contribute to GA inactivation. That the *NCED* gene, which contributes to ABA biosynthesis (Finkelstein and Rock, 2002), is down-regulated by GA treatment (Figure 5G) also indicates that GA could affect ABA production. We suppose that this latter antagonistic effect of GA and ABA on each other's biosynthesis, in addition to their antagonistic effects on hormone signaling, contributes directly to the rapid responses in petal to the presence of both hormones.

Putative crosstalk nodes coupling diverse hormone-associated pathways were also identified in this study. *PAO5* (GACN01003808) and *MMS21* homologs (GACN01025802), involved in the cytokinin signaling pathway (Brenner et al., 2005; Huang et al., 2009), respond to GA and ABA antagonistically (Supplemental Table S8). The homolog of a member of the PP2C family (GACN01010139), which is predicted to be enriched during an ethylene- and auxin-induced response or jasmonic acid- and salicylic acid-mediated signaling (Heyndrickx and Vandepoele, 2012), is up-regulated by GA but down-regulated by ABA (Supplemental Table S9). Other hormone pathways, including CK, ET, brassinosteroid (BR), JA and salicylic acid (SA) signaling, are also altered by GA and/or ABA (Supplemental Table S5). These data, in accordance with the report by Nemhauser et al. (2006), support the hypothesis that *G. hybrida* petal growth is regulated by a substantial network of interconnected hormonal pathways and feedback circuits.

### DEGs involved in transcriptional regulation, apoptosis and cell wall organization

Our data show that cell expansion is critical for petal growth at later stages. GO-BP enrichment analyses for Class III DEGs at 12 h show that the category “cell wall organization” especially the sub-category “cell wall loosening” is overrepresented (Table 2), and the DEGs involved, encoding a group of expansins, are activated by GA, but repressed by ABA. Expansins are considered to be molecular markers of cell elongation (Bai et al., 2012; Ikeda et al., 2012). The enrichment of these genes after 12 h hormone treatment is consistent with the notion that GA-induced cell wall loosening contributes to cell elongation during petal growth.

Analysis of the Class II DEGs indicated that the GO category “apoptosis” changes rapidly during the 10 h interval between the two sampling periods, apparently in a GU/AD pattern (Table 2). As explored further below, this suggests an intriguing relationship between petal development and apoptosis during GA and ABA treatment. BlastX searches indicated that the genes involved code for a group of pathogen-associated disease resistance proteins. No clear association between these proteins and petal development has yet been described, but a TIR-NB-LRR protein (Kim et al., 2012) provides one example where disease resistance proteins contribute to plant development, suggesting that these proteins are functional in diverse biological processes. On the other hand, it is well established that pathogen-induced plant immunity is regulated by GA and ABA. For example, ABA can induce plant immunity-associated callose deposits by which an efficient pathogen-resistant barrier is formed (Luna et al., 2011). Mutations in the genes involved in ABA biosynthesis or signaling can enhance resistance to some pathogens (Sánchez-Vallet et al., 2012). It is also evident that GA and DELLA proteins are linked to disease responses and the associated cell wall modification (De Bruyne et al., 2014). Apoptosis is a critical event during plant development, as well as in pathogen-induced plant immunity. For example, vascular development in the plant coordinates the different phases of xylem maturation, including secondary wall formation, cell death and other processes (Bollhoner et al., 2012). Cell death is included in the hypersensitive responses induced by pathogens in the plant (Lam et al., 2001). However, the contribution of apoptosis to GA/ABA-regulated petal development remains to be elucidated.

The third group of DEGs enriched in GA- and ABA-treated petals is involved in regulation of transcription. There were nine TFs that appeared after 2 h treatment and showed a GD/AU pattern (Table 2). The homologs of some of these proteins are reported to be regulated by GA and ABA in other plant species. For example, the *Arabidopsis* homolog of the putative *G. hybrida MYB62* (GACN01007389) plays a role in GA biosynthesis and signaling pathways. Overexpression of the *AtMYB62* gene results in a GA-deficient phenotype that can be partially reversed by exogenous application of GA (Devaiah et al., 2009). The *G. hybrida* DEG, GACN01021753, encodes a putative member of the homeodomain leucine zipper (HD-Zip) family. Overexpression of some members of the HD-Zip family in *Arabidopsis* and *Oryza sativa* (rice) affects organ elongation and expansion by modulation of GA and/or ABA metabolism and signaling (Agalou et al., 2008; Son et al., 2010). The DEG GACN01038219 encodes a putative WRKY protein. Two WRKY homologs in rice, OsWRKY51 and OsWRKY71, interact in aleurone cells and establish a novel mechanism of crosstalk between ABA and GA signaling (Xie et al., 2006). In addition, we found that GACN01018830 codes for a homolog of the TCP-domain containing protein, CYCLOIDEA-like 5 (CYC5). CYC homologs are reported to be involved in flower symmetry regulation in many plant species (Martin-Trillo and Cubas, 2010). In *G. hybrida*, genes for CYC homologs were also identified previously (Broholm et al., 2008; Tahtiharju et al., 2012). Expression analysis during ray flower ligule development indicates that *GhCYC3* contributes to early petal growth and correlates with cell division, while *GhCYC5* is instead activated at late stages when elongation growth is ceasing (Kotilainen et al., 1999; Juntheikki-Palovaara et al., 2014). Integrating the previously reported findings with our data in the current study, we conclude that these various transcription regulators contribute to cell growth in the petal of *G. hybrida*. Under the influence of GA, genes involved in “cell wall loosening” and “apoptosis” are released from transcriptional repression by these TFs. ABA, on the contrary, activates these TF genes and result in repression of cell elongation.

In summary, we have identified a group of DEGs from the basal region of the petals of *G. hybrida* that show antagonistic transcription profiles during GA and ABA treatment. Annotation enrichment analyses further clarified the biological processes and pathways involved, as well as the co-targets for both hormones. Our data support the hypothesis that cell expansion in *G. hybrida* petals at inflorescence stage 3 is attributed to the regulation of transcription and apoptosis, which consequently lead to activation of cell wall loosening. GA and ABA work antagonistically to balance the responses to developmental signals and guarantee the smooth running of this network.

### Conflict of interest statement

The authors declare that the research was conducted in the absence of any commercial or financial relationships that could be construed as a potential conflict of interest.
